# Does Healthcare Provider Counseling for Weight Management Behaviors among Hispanic Adults Who Are Overweight/Obese Vary by Acculturation Level?

**DOI:** 10.3390/ijerph20042778

**Published:** 2023-02-04

**Authors:** Mary L. Greaney, Furong Xu, Christie L. Ward-Ritacco, Steven A. Cohen, Kerri A. Ellis, Deborah Riebe

**Affiliations:** 1 Department of Health Studies, University of Rhode Island, Kingston, RI 02881, USA; 2 School of Education, University of Rhode Island, Kingston, RI 02881, USA; 3 Department of Kinesiology, University of Rhode Island, Kingston, RI 02881, USA; 4 College of Nursing, University of Rhode Island, Kingston, RI 02881, USA

**Keywords:** acculturation, NHANES, obesity, health care provider counseling

## Abstract

This cross-sectional study explored differences in the receipt of health care provider (HCP) counseling to control/lose weight and adopt weight-related lifestyle behavior changes among Hispanic respondents according to acculturation level. Differences in reported action regarding HCP counseling were also examined. Data from four National Health and Nutrition Examination Survey (NHANES) cycles (2011–2018) were analyzed, with the analytic sample limited to Hispanic respondents who were overweight/obese. Respondents’ acculturation levels were derived from their reported country of origin and the primary language spoken at home. Respondents who reported speaking only Spanish or more Spanish than English at home were classified as primarily speaking Spanish at home. In contrast, those who reported speaking Spanish and English equally, more English than Spanish, or only English were categorized as primarily speaking English at home. Weighted multivariate logistic regression models were utilized to calculate adjusted odds ratios (ORs) and 95% confidence intervals (CIs) to determine if differences in acculturation levels existed regarding the likelihood of receiving HCP counseling to (1) control/lose weight, (2) increase exercise/PA, and (3) reduce fat/calorie intake. Similar analyses examined differences in reported action regarding HCP counseling according to acculturation level. The analysis found no significant differences in receiving HCP counseling according to acculturation level. However, non-US-born respondents who primarily spoke Spanish at home were less likely than US-born respondents to report acting to control/lose weight (*p* = 0.009) or increase exercise/PA (*p* = 0.048), but were more likely to report having taken action to reduce fat/calorie intake (*p* = 0.016). This study revealed differences between acting on recommendations of health care professionals according to acculturation level, indicating a need for interventions tailored to acculturation levels.

## 1. Introduction

Approximately 18.5% of the United States (US) population identifies as Hispanic/Latino [1], and the number of people identifying as Hispanic/Latino is expected to nearly double by 2060 [2]. Almost half of all immigrants (44%, 19.8 million) living in the US report being of Hispanic origin [3], making this population the second fastest-growing minority population [4]. There are well-documented disparities in overweight/obesity by ethnicity, with 79.6% of Hispanics/Latinos being overweight or having obesity compared to 70.9% of non-Hispanic whites [5]. The prevalence of obesity is approximately 10 percentage points higher among Hispanics/Latinos (referred to as Hispanics, from this point forward) than non-Hispanic white adults (48% vs. 38%) [6]. As the health risks of obesity include an increased risk of some cancers [7], diabetes [8], heart disease [9], and mortality [10,11], disparities in obesity present a significant public health challenge.

Immigration to the US can result in changes in health-related behaviors [12,13]. Acculturation is a multi-dimensional process in which immigrants may adopt new beliefs, practices, and behavior of the country they immigrated to, while retaining the culture, values, and beliefs of their country of origin [14]. Despite some potential benefits associated with acculturation (e.g., increased leisure-time physical activity (PA) and exercise) [12,13], research with Hispanic immigrants in the US has found that greater acculturation is associated with decreased diet quality [15,16,17,18], increased sedentary behaviors [12,13], and risk of obesity [19,20,21]. Due to the higher prevalence of obesity among Hispanic adults, the health risks of obesity, and the increasing number of Hispanic immigrants in the US, it is essential to understand factors that may promote changes in obesity-related health behaviors among Hispanics in the US.

Discrimination based on race or ethnicity is one of the most frequent types of discrimination reported by patients in the US [22]. As such, Hispanic patients may experience negative interactions with health care providers (HCPs) and face discrimination when accessing health care [23,24,25]. A study with English-speaking adults found that 22.9% of Hispanic participants reported experiencing discrimination in the healthcare system due to their ethnicity [22]. Similarly, an analysis of data from the California Health Information Survey found that 20.5% of Hispanic respondents reported facing discrimination in healthcare due to their ethnicity. Moreover, 11.8% encountered discrimination due to language/accent, and 21.5% due to their type of insurance [26]. Respondents who reported discrimination due to race/ethnicity had lower attendance at preventive care services [26]. Specifically addressing interactions with HCPs, a recent analysis of data from the Survey of California Adults on Serious Illness and End-of-Life 2019 found that 11% of Hispanic respondents reported feeling discrimination from an HCP due to their ethnicity, while 10% reported encountering discrimination due to their language [27]. Experiencing discrimination due to racial/ethnic background and language is associated with increased medical mistrust [27,28]. Research providing evidence of Hispanic patients facing discrimination in the US is important, as it indicates the need for intervention among HCPs and staff at related facilities, as only one-third (37%) of non-US-born Hispanics living in the US are proficient in English (speak only English at home or rate themselves as speaking English very well) [4].

Among all patients, physician counseling has been found to increase an individual’s intention to control and/or lose weight [29,30]. Body-weight related counseling should include discussions about caloric intake and expenditure, given their importance to energy balance. It should also address PA due to its association with weight gain prevention and maintenance of weight loss [31,32]. Brief counseling by physicians (3–5 min/session) can increase PA [33]. Nonetheless, research indicates that weight loss and dietary counseling by HCPs (e.g., physicians, nurse practitioners, physician assistants) during medical visits often does not occur [34]. Research has found that Hispanic adults are more likely to receive dietary [34] and PA counseling than white adults [35,36]. However, only limited research has examined differences in diet and exercise/PA counseling according to acculturation [37]. This research is needed, given the risk of obesity among Hispanics and the increasing number of Hispanic immigrants in the US.

There is a lack of research exploring weight-related counseling by HCPs to determine if there is a difference in HCP counseling for weight loss and associated lifestyle behaviors according to acculturation level. This research is needed due to the disparities in overweight and obesity by ethnicity among US adults and the benefits of HCP counseling. Moreover, research is needed to determine if patients who received HCP counseling act on the advice they received. Thus, the present study, as an analysis of nationally representative data, was designed to explore (1) differences in the receipt of HCP counseling to control/lose weight and adopt weight-related lifestyle behavior changes among Hispanic respondents by acculturation level and (2) differences in reported action according to acculturation level regarding the recommendations of health care providers.

## 2. Materials and Methods

This study was a cross-sectional analysis of respondents 18 years or older from four National Health and Nutrition Examination Survey (NHANES) data cycles collected between 2011 and 2018 (*n* = 23,825). To enroll a representative sample of the US civilian, non-institutionalized population, NHANES utilizes multi-stage probability [38]. The sample for this study was limited to respondents with a body mass index (BMI) of 25 kg/m^2^ or higher who reported seeing an HCP (excluding hospital stays, emergency room, home visits, and telephone calls) at least once in the 12 months before data collection (*n* = 13,158). Measured height and weight were used to calculate BMI, which was then used to determine weight status [overweight (25–29.9 kg/m^2^) or obese (≥30 kg/m)]. The analytic sample was then further limited to respondents who self-identified as Hispanic, Mexican American, or as part of another Hispanic group, with data on their primary language spoken at home, leaving an analytic sample of 3263. The study was approved by the Institutional Review Board (IRB) at the University of Rhode Island and classified as exempt (IRB#: 122-008).

### 2.1. Measures

Demographics: respondents reported their age, self-identified gender (male, female), marital status (married/living with a partner, widowed, divorced/separated, never married), educational attainment (high school diploma or less, some college or above), and smoking status (never, former, current) [38]. Three age groups were created (18–39, 40–64, 65+ years of age). Family income and family size were used for calculating the poverty-to-income ratio (PIR), which is the ratio of family income to the federal poverty level. The PIR was then dichotomized to at/above or below the poverty level.

Acculturation: NHANES respondents reported their place of nativity, dichotomized as being non-US-born (including US territories) or US-born (all 50 states and Washington, DC) [38]. Respondents also reported the language they usually speak at home (only Spanish, more Spanish than English, both equally, more English than Spanish, or only English). Respondents who reported speaking only Spanish or more Spanish than English at home were classified as primarily speaking Spanish at home, while those who reported speaking both Spanish and English equally, more English than Spanish, or only English were categorized as primarily speaking English at home. Respondents who spoke English and Spanish equally at home were categorized as primarily speaking English, since English language proficiency influences healthcare access and quality [39], and language proficiency for these individuals would be less likely to be a barrier to care [40]. These two items were used to create three acculturation categories/levels: (1) US-born, (2) non-US-born + primarily speaks Spanish at home, and (3) non-US-born + primarily speaks English at home. The use of language spoken at home as a proxy measure for acculturation has been used previously with Hispanic respondents [41,42].

HCP counseling: three items from the NHANES medical conditions survey assessed the receipt of HCP counseling. Respondents reported whether, in the past 12 months, a doctor or health professional had recommended (yes, no) that they: (1) control/lose weight, (2) reduce fat/calorie intake to lower their risk for certain diseases, and (3) increase exercise/PA [38].

Action on HCP counseling: respondents who reported receiving HCP counseling to control/lose weight were asked if they had acted on the advice provided by the health care professional (yes, no). Similarly, respondents who reported being counseled to increase exercise/PA or reduce fat/caloric intake reported whether they had acted on the HCP counseling received [38].

### 2.2. Data Analysis

All analyses were weighted according to the guideline for the analysis of data from multiple NHANES survey cycles [43]. Sample characteristics were expressed as frequencies and weighted proportions [n (weighted %)] for the categorical variables and weighted mean ± standard errors for continuous variables. Chi-square or t-tests were conducted to determine if there were differences in the demographic characteristics regarding weight status for categorical and continuous variables, respectively. Next, weighted multivariate logistic regression models were utilized to calculate adjusted odds ratios (ORs) and 95% confidence intervals (CIs) to determine if differences existed according to acculturation level regarding the likelihood of receiving HCP counseling to (1) control/lose weight, (2) increase exercise/PA, and (3) reduce fat/calorie intake. The US-born group served as the reference group. We adjusted all models for the covariates (age, gender, education level, PIR, and marital status), which were added to the model simultaneously. We then constructed a second series of weighted multivariate logistic regression models to examine differences in reported action regarding HCP counseling according to the acculturation level among those whose HCP recommended action. Again, all models included the covariates (age, gender, education level, PIR, and marital status). The analysis was conducted using SAS 9.4 (SAS Institute Inc., Cary, NC, USA) with *p* < 0.05 set a priori for statistical significance.

## 3. Results

Most respondents were female (55.1%), married or living with a partner (67.7%), or were obese (56.5%). More than half (58.2%) of the respondents had a high school diploma or lower level of educational attainment. In addition, 42.1% of respondents were non-US-born and primarily spoke Spanish at home, 14.0% were non-US-born and primarily spoke English at home, and 43.9% were US-born. As seen in Table 1, there was a difference in weight status by gender, PIR, and age. More women than men (58.1% vs. 41.9%) were classified as obese (*p* < 0.001). Among individuals aged 65+, a greater percentage were overweight than obese (13.5% vs. 11.3%, *p* = 0.011), while a greater percentage of respondents with a PIR ≥ 1.0 were overweight versus obese (75.4% vs. 71.4%. *p* = 0.020).

**Figure 1:**
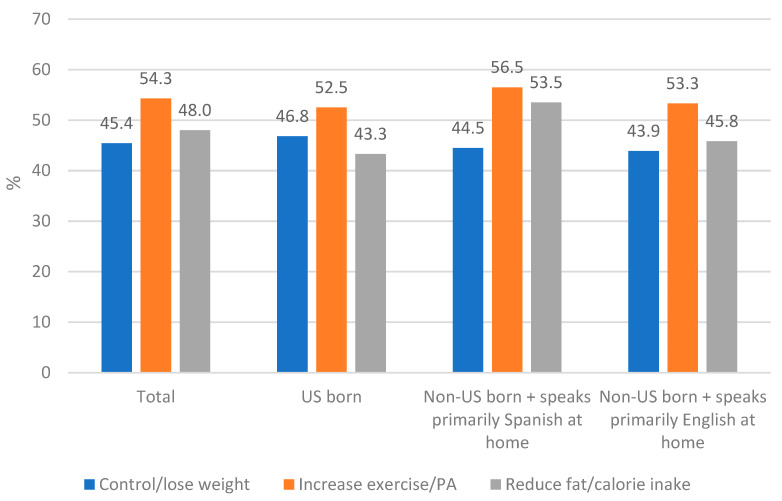
Receipt of healthcare provider counseling according to acculturation level.

In total, 45.4% of respondents reported receiving HCP counseling to control/lose weight, 48.0% to reduce fat/calorie intake, and 54.3% were counseled to increase exercise/PA (See Figure 1). As seen in Table 2, the logistic regression analysis determined that there was no statistically significant difference in the likelihood of receiving HCP counseling to control/lose weight, increase exercise/PA, and reduce fat/calorie intake according to acculturation group (US-born, non-US-born + primarily speaks Spanish at home, and non-US-born + primarily speaks English at home).

**Figure 2:**
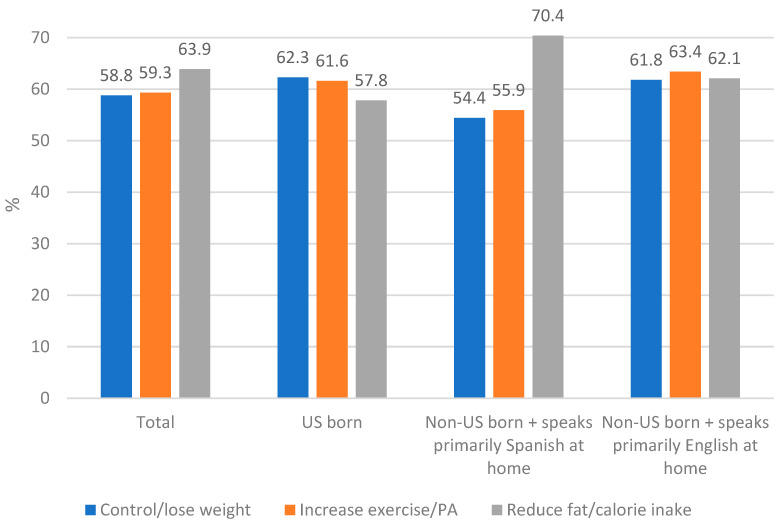
Percent of respondents who reported taking action regarding health care provider counseling.

As seen in Figure 2, 58.8% of respondents who received HCP counseling reported taking action to control/lose weight, while 63.9% reported reducing fat/calorie intake, and 59.3% reported increasing exercise/PA. A greater percentage (63.9%) of respondents reported acting to reduce fat/calorie intake. The results of the logistic regression analyses determined that respondents who were non-US-born and primarily spoke Spanish at home were less likely to have acted to control/lose weight (OR = 0.69, 95% CI: 0.52–0.91) or to increase exercise/PA (OR = 0.77, 95% CI 0.60–1.00) than US-born respondents. However, as seen in Table 3, respondents who were non-US-born and primarily spoke Spanish at home were more likely than US-born respondents to have taken action to reduce fat/calorie intake (OR = 1.43, 95% CI: 1.07–1.90). There were no significant differences between US-born and non-US-born respondents who primarily spoke English at home regarding reported action regarding the three assessed behaviors (See Table 3).

## 4. Discussion

This analysis of 2011–2018 NHANES data found no statistically significant differences in receiving HCP counseling according to acculturation level among Hispanic respondents who were overweight or obese. This is a positive finding, which could possibly be the result of efforts to increase cultural competency among HCP and healthcare organizations [44]. Nonetheless, it is concerning that in the current study of US adults who self-identified as Hispanic, Mexican America, or as being part of another Hispanic group and who were overweight/obese, less than half received HCP counseling to lose weight (45.4%) or reduce fat/calorie intake (48%), while 54.3% were advised to increase exercise/PA. Although not assessed in this study, prior research has found that Spanish-speaking patients with language-concordant physicians were more likely to receive counseling on diet and PA than patients with language-discordant physicians; however, after controlling for covariates, there was no significant difference in PA counseling [45]. The importance of exercise/PA counseling during all healthcare contacts, particularly in primary care, should not be understated, as there are significant health benefits of PA/exercise, independent of changes in weight [46,47,48,49].

In the current study, about 60% of respondents who received HCP counseling reported acting on this advice (58.8% to control/lose weight, 63.9% to reduce fat/calorie intake, 59.3% to increase exercise/PA). Respondents who were non-US born and spoke only Spanish or more Spanish than English at home were less likely to report acting on HCP counseling for weight loss/control and exercise/PA and more likely to take action for reducing fat/calorie intake. An analysis of national data found that among US adults with arthritis who received an HCP recommendation to attend a self-management class/workshop, 52% of those advised attended a workshop, suggesting that HCP advice can motivate action [50]. Although this percentage is smaller than that identified in the current study, it suggests that HCP counseling that provides a specific recommendation (e.g., attending an evidence-based program) could increase reported action for changing behavior. Expanding the use of the five As (ask, advise, agree upon, assist, arrange follow-up) for obesity management by HCPs, including medical assistants and community health workers, may increase the incidence of patients acting on HCP advice. These guidelines would have HCPs ask about or assess the patient’s willingness to change [51]. HCP counseling should address possible barriers to adopting healthful behaviors, including lack of time, and focus on increasing self-efficacy. Using interactive behavior change technologies, such as voice response telephone calls or self-monitoring apps that provide feedback, could help facilitate behavior change, if integrated into the medical setting [52], while overcoming limitations of medical appointments (e.g., time constraints).

Interestingly, differences in reported action by the acculturation group were identified, perhaps recognizing the need for culturally-specific recommendations related to weight control and PA. Respondents who were non-US-born and primarily spoke Spanish at home were less likely to have taken action to control/lose weight or increase exercise/PA than US-born respondents. Unfortunately, the NHANES survey did not assess reasons for not acting on HCP counseling. However, it is possible that individuals with lower levels of acculturation may have lower incomes and face greater barriers to increasing exercise/PA, including working multiple jobs, living in areas without parks, and safety concerns. PA during work or commuting may also deter leisure-time PA. A separate analysis of NHANES data determined that foreign-born Mexican Americans living in the US participated in less leisure time PA/week, but more PA at work and during commuting than US-born Mexican Americans [53].

Conversely, respondents who were non-US-born + primarily spoke Spanish at home were more likely than US-born respondents to have taken action to reduce fat/calorie intake. The reasons for this difference are unknown and should be explored in future research. Research has found that Hispanics with low levels of acculturation may access information differently and have different needs than Hispanics with higher levels of acculturation [54,55]. The use of remote peer mentors to encourage behavior change has been found to promote behavior change among patients with diabetes receiving care in a safety net healthcare delivery setting [56]. This strategy could be explored in future research.

Many respondents reported not acting on their HCPs’ recommendations. To address this, HCPs should discuss evidence-based strategies for behavior change with patients. This may require additional training for HCPs and could also be done by other members of healthcare teams, including medical assistants. Patients should also be provided with appropriate referrals to promote increased adoption of offered recommendations, including community education programs, dietitians, and exercise physiologists. Evidence-based interactive behavior change technologies incorporated into the medical appointment and health care setting [52] could also be explicitly tailored for these racial/ethnic groups. The use of mHealth technology, including tablets, mobile apps, and wearable devices (e.g., smart watches), is being used to promote healthy diets and PA [57]. These interventions show promise, but barriers to their use must be addressed. For example, a small pilot study with Hispanic men (n = 18) found that lack of technology literacy and unreliable internet service were barriers to using a FitBit (step counting device) and logging weight on a smart scale [58].

The study has several limitations that should be noted, including the cross-sectional study design, reliance on self-reported data regarding the receipt of HCP counseling, and reported action on this counseling. Weight status, however, was calculated using measured height and weight, which is highly recommended due to known underestimates of weight and overestimates of height when these values are self-reported. The acculturation measure used in the present study is centered on language acculturation; therefore, the use of more detailed measures, such as the Short Acculturation Scale for Hispanics [59] or the Bidimensional Acculturation Scale for Hispanics [60], may have yielded different results and may be appropriate for use in future studies. Nonetheless, the use of language at home as a proxy measure for acculturation has been used previously with Hispanic respondents [41,42]. It is estimated that 60% of non-US-born Hispanics in the US speak Spanish at home, 35% speak both English and Spanish, and 5% speak English [61].

It would be advantageous if future versions of the NHANES survey assessed information about HCPs’ language or ethnic/language concordance with the respondents, as this detail would benefit research in this and related areas. Additionally, the NHANES survey does not assess the scope and frequency of HCP counseling, which may play an essential role in the adoption of positive lifestyle behavior change. There is also the possibility of response bias, with respondents being reluctant to report that they did not receive counseling and/or did act on recommended advice, as well as selection bias, with undocumented Hispanic persons in the US choosing not to participate in the NHANES survey. Lastly, Hispanics in the US are heterogenous, and this analysis does not recognize this heterogeneity.

The study has several strengths. It is one of the first studies to explore differences in HCP counseling and reported action according to acculturation level among Hispanic adults in the US. In addition, the sample is drawn from a national dataset. Therefore, the results are generalizable to non-institutionalized civilian adults aged 18+ in the US who identify as being Hispanic, Mexican American, or as being part of another Hispanic group and who are overweight/obese according to the NHANES’ sampling frame.

## 5. Conclusions

The current study found no differences in the receipt of HCP counseling according to acculturation level among Hispanics included in the NHANES sample, which is a positive finding. However, only about 60% of respondents reported acting on HCP recommendations, and differences in reported action on HCP counseling were identified by acculturation level. Non-US-born respondents who primarily spoke Spanish at home were less likely to report acting to control/lose weight or increase exercise/PA, but were more likely to report having taken action to reduce fat/calorie intake than US-born respondents. These differences should be explored in further research, with more nuanced measures of acculturation and with more detailed assessments of HCP counseling. The findings also clearly indicate the need for increased HCP counseling for weight management, PA, and diet among Hispanic adults in the US who are overweight or obese, regardless of acculturation level. HCPs likely need additional acculturation level-specific training regarding addressing behavior change, including overcoming barriers to change, to help patients act on offered advice to address the growing obesity and physical inactivity epidemic among US adults.

## Figures and Tables

**Table 1:** Characteristics of respondents aged 18+ stratified by weight status, NHANES 2011–2018.

Variable	Total n = 3263	Overweight n = 1422 (43.5%)	Obese n = 1841 (56.5%)	*p*-Value
Gender				
Male	1393 (44.9)	681 (48.7)	712 (41.9)	<0.001 *
Female	1870 (55.1)	741 (51.3)	1129 (58.1)	<0.001 *
Age (years), weighed mean ± standard errors	43.8 ± 0.5	44.1 ± 0.5	43.5 ± 0.6	0.389
Age groups				
18–39 years	999 (43.8)	437 (44.6)	562 (43.3)	0.546
40–64 years	1538 (43.9)	637 (41.9)	901 (45.5)	0.063
65+ years	726 (12.2)	348 (13.5)	378 (11.3)	0.011 *
Education level				
High school or less	1973 (58.2)	872 (57.6)	1101 (58.6)	0.612
College or above	1142 (41.8)	478 (42.4)	664 (41.4)	0.612
PIR ≥ 1.0	2018 (73.1)	896 (75.4)	1122 (71.4)	0.020 *
Marital status				
Married or living with partner	2070 (67.7)	905 (68.6)	1165 (67.0)	0.421
Windowed	208 (3.9)	89 (3.9)	119 (3.9)	1.000
Divorced/separated	478 (13.3)	204 (12.8)	274 (13.7)	0.390
Never married	363 (15.0)	154 (14.6)	209 (15.4)	0.683
Place of nativity				
US-born	1297 (43.9)	475 (37.0)	822 (49.2)	<0.001 *
Non-US-born	1966 (56.1)	947 (63.0)	1019 (50.8)	<0.001 *
Length of time living in US				
<10 years	243 (16.6)	132 (18.7)	111 (14.5)	0.060
10–<20 years	473 (32.1)	231 (33.5)	242 (30.8)	0.279
≥20 years	1123 (51.4)	521 (47.8)	602 (54.7)	0.021 *
Acculturation				
US-born	1297 (43.9)	475 (37.0)	822 (49.2)	<0.001 *
Non-US-born + speaks primarily Spanish at home	1523 (42.1)	748 (48.7)	775 (37.0)	<0.001 *
Non-US-born + speaks primarily English at home	443 (14.0)	199 (14.3)	244 (13.8)	0.733

Note: Respondents’ characteristics were expressed as n (weighted %), unless otherwise specified; BMI = body mass index, overweight = BMI classified as 25.0 to <30 kg/m^2^; obese = BMI is 30.0 kg/m^2^ or higher, PIR = poverty-to-income ratio; * symbol indicates that the *p*-value is significant (*p* < 0.05).

**Table 2:** Comparative odds ratios of receiving health care provider (HCP) counseling by acculturation level.

	HCP Counseling to					
	Control/Lose Weight		Increase Exercise/PA		Reduce Fat/Calorie Intake	
	Adjusted OR (95% CI)	*p* Value	Adjusted OR (95% CI)	*p* Value	Adjusted OR (95% CI)	*p* Value
US-born	REF		REF		REF	
Non-US-born + speaks primarily Spanish at home	0.82 (0.62, 1.09)	0.165	1.04 (0.83, 1.30)	0.752	1.26 (0.95, 1.67)	0.105
Non-US-born + speaks primarily English at home	0.81 (0.58, 1.12)	0.193	0.95 (0.74, 1.22)	0.660	1.04 (0.76, 1.43)	0.804

Note: The adjusted odds ratio (OR) with 95% confidence interval (CI) was obtained by performing PROC SURVEYLOGISTC, adjusted for age, sex, education level, PIR, and marital status.

**Table 3:** Comparative odds ratios for taking action on health care provider counseling according to acculturation level.

	Reported Action to					
	Control/Lose Weight		Increase Exercise/PA		Reduce Fat/Calorie Intake	
	Adjusted OR (95% CI)	*p* Value	Adjusted OR (95% CI)	*p* Value	Adjusted OR (95% CI)	*p* Value
US-born	REF		REF		REF	
Non-US-born + speaks primarily Spanish at home	0.69 (0.52, 0.91)	0.009 *	0.77 (0.60, 1.00)	0.048 *	1.43 (1.07, 1.90)	0.016 *
Non-US-born + speaks primarily English at home	0.91 (0.62, 1.35)	0.644	1.16 (0.83, 1.62)	0.371	1.15 (0.75, 1.76)	0.520

Note: The adjusted odds ratio (OR) with 95% confidence interval (CI) was obtained by performing PROC SURVEYLOGISTC, adjusted for age, gender, education level, PIR, and marital status. * Indicates a *p*-value of <0.05.

## Data Availability

The data used for the current study were publicly available on the official Centers for Disease Control and Prevention and the National Center for Health Statistics website: https://wwwn.cdc.gov/nchs/nhanes/sasviewer.aspx, accessed 21 May 2021.
